# Supplementary material for: Comprehensive Annotation and Functional Exploration of MicroRNAs in Lettuce
Source: Front Plant Sci. 2021 Dec 24;12:781836. doi: 10.3389/fpls.2021.781836 (PMC8739914; doi:10.3389/fpls.2021.781836)
Supplement: Supplementary file 3 [file Data_Sheet_9.ZIP › GSM2230752.plot/Lsa-miRN21_Lsat_1_v5_gn_5_53081.1_1634_TPlot.pdf]

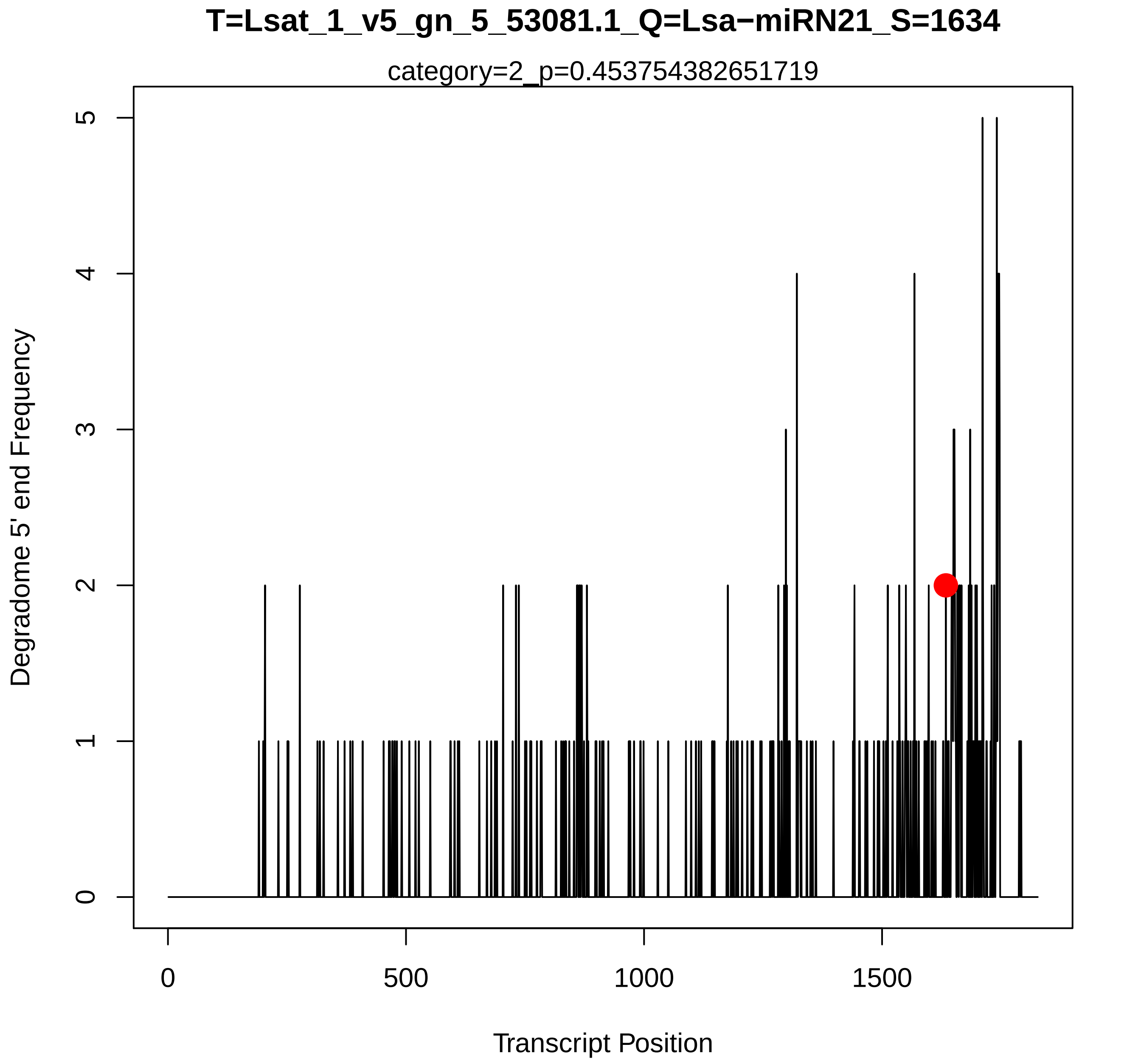

T=Lsat_1_v5_gn_5_53081.1_Q=Lsa−miRN21_S=1634
category=2_p=0.453754382651719
Degradome 5' end Frequency
0
1
2
3
4
5
0
500
1000
1500
Transcript Position
